# Gypsum-Based Humidity-Control Material: Preparation, Performance and Its Impact on Building Energy Consumption

**DOI:** 10.3390/ma16155211

**Published:** 2023-07-25

**Authors:** Xi Li, Maoyu Ran

**Affiliations:** 1 School of Architecture, Huaqiao University, Xiamen 361021, China; 2 Xiamen Key Laboratory of Ecological Building Construction, Xiamen 361021, China

**Keywords:** humidity control, composite material, energy saving simulation, gypsum

## Abstract

This paper introduces a new type of gypsum-based humidity-control material. The material combines gypsum–silica gel humidity-control material with 20% sepiolite powder activated by calcium chloride. Both experimental and simulation studies were conducted to assess its humidity-control performance. The experimental results indicate that gypsum-based humidity-control material has the property of absorbing moisture in high-humidity environments and releasing moisture in low-humidity environments. Moreover, both environmental temperature and relative humidity (RH) have an impact on the material’s humidity-control performance. At a relative humidity of 97.4%, the maximum equilibrium moisture content of the material is 0.225 g/g, which is 1.4 times that of the gypsum–silica gel humidity-control material and 4.5 times that of pure gypsum material. The simulation results indicate that gypsum-based humidity-control material effectively mitigates indoor relative humidity fluctuations and maintains indoor air relative humidity within a narrow range. Furthermore, the material has the potential to reduce building energy consumption. This is especially evident under climate conditions with large temperature and relative humidity differences between day and night, such as in Beijing, Paris, and Atlanta. The maximum potential energy-saving rate in Beijing can reach up to 19.31%.

## 1. Introduction

The indoor environment plays a crucial role in determining individuals’ physical and mental well-being, a relationship that is primarily affected by indoor environmental parameters [1,2,3]. Relative humidity is one such parameter that significantly influences thermal comfort, building load, indoor air quality, and occupant work efficiency [4,5]. To create a comfortable indoor environment, it is pivotal to regulate relative humidity based on active or passive methods. The former involves using heat, ventilation, and air conditioning systems to either humidify or dehumidify indoor air, thereby keeping relative humidity at cozy levels for occupants. However, this method consumes energy and causes environmental pollution, undermining China’s strategy for renewable energy development. Reports indicate an average annual growth rate of 5.39% in China’s energy consumption during building operations from 2005 to 2018 [6]. In contrast, the latter method utilizes renewable energy or materials with moisture absorption and desorption capabilities. This approach aims to achieve the same goal of enhancing comfort while reducing building energy consumption [7,8,9]. This ecological approach holds great significance for improving indoor living space and building energy conservation.

As a passive energy-saving technology for regulating indoor humidity, humidity-control materials stand out among numerous passive technologies. The concept of humidity-control materials was initially proposed by Nishito and Miyano in Japan in the 1940s [10]. It refers to the method of automatically regulating indoor air humidity without relying on energy-consuming equipment, based on the moisture absorption and desorption characteristics of the material. It is acknowledged as an environmentally benign passive control method. It can manage indoor relative humidity fluctuations and maintain indoor relative humidity within a comfortable range, thereby avoiding negative impacts on human health caused by high or low relative humidity environments [11,12].

In recent years, the utilization of humidity-control materials for regulating indoor relative humidity has yielded promising results [13,14,15]. Zhang’s experimentation with humidity-control materials placed in an artificial climate chamber indicated their effectiveness in regulating indoor humidity levels [16]. Ge’s inquiry focused on the moisture buffering performance of common humidity-control materials. The results revealed significant variations in the moisture absorption and desorption capabilities across similar materials [17]. Simonson’s numerical simulation study demonstrated that incorporating humidity-control materials in indoor spaces led to a reduction in required ventilation rates. Importantly, this reduction was achieved without compromising comfort or air quality [18]. Zhang’s application of numerical simulation techniques revealed the significant impact of humidity-control materials on building energy consumption in different climatic conditions. This study highlights their potential for up to 25% energy savings in temperate and semi-arid climates [19].

Currently, various types of humidity-control materials have been developed and classified as biomass, organic, inorganic, and composite materials [20]. Biomass materials have relatively large moisture capacity, but they exhibit smaller vapor permeability and slower moisture transfer rate [21]. Organic materials can absorb moisture hundreds of times their own weight, but they have weaker desorption ability [22]. Inorganic materials possess open porous structures and strong adsorption capabilities. Among them, gypsum is a widely used lightweight construction material known for its cost-effectiveness and eco-friendly production process. Its high porosity and uniform pore size distribution enable favorable permeability, making it suitable for humidity-control applications. Roel investigated the moisture absorption and desorption performance of gypsum boards coated with latex paint compared to bare gypsum boards. The findings indicated that applying latex paint on the surface of gypsum boards significantly diminishes their moisture buffering capacity [23]. Zhang evaluated the humidity-control capabilities of magnesite board, diatomaceous earth, and gypsum board. The results revealed that they all possess varying degrees of humidity-control capabilities, with magnesite board performing the best, followed by diatomaceous earth, and gypsum board performing poorly [24]. Shahrzad found that placing gypsum inside concrete walls under higher indoor ventilation rates can maintain indoor relative humidity at approximately 60% [25]. Although gypsum possesses certain moisture control capabilities, it presents two limitations in terms of its moisture absorption and desorption performance. Firstly, within the relative humidity range of 40–70%, gypsum exhibits relatively low equilibrium moisture content, which fails to meet the demand for substantial moisture absorption within the standard humidity range. Secondly, gypsum displays a slow rate of moisture absorption and desorption, making it challenging to promptly respond to dynamic humidity changes.

To enhance the humidity-control performance of gypsum, numerous researchers have chosen gypsum as the matrix and incorporated other materials to develop gypsum-based composite materials [26,27]. Jiang employed sepiolite powder activated by calcium chloride as an additive and incorporated it into gypsum to prepare a composite material. The adsorption and desorption performance were tested, revealing that the adsorption and desorption capacity of the samples increased with the increase in the dosage of activated sepiolite powder. However, with a continuous increase in the content of activated sepiolite powder, wetting phenomena were observed on the sample surface. Consequently, the optimal additive content was found to be 20% of activated sepiolite powder [28]. Shang introduced lithium chloride into gypsum and developed a novel material. The research indicated that this material exhibited good humidity absorption and desorption performance, with a maximum moisture absorption capacity of 0.410 g/g [29]. Lee added activated clay into gypsum to fabricate a new gypsum-based composite material. The experimental results showed that the humidity absorption and desorption performance of the material improved with an increasing amount of clay addition, reaching its peak when the clay content reached 70% [30]. Shang successfully developed a gypsum-based humidity-control material by mixing it with adsorbent materials such as plant fiber, kaolin, and activated carbon [31].

In conclusion, gypsum is a traditional building material with humidity-control properties, but it has limitations in terms of moisture absorption and desorption performance. The current focus of research lies on composite humidity-control materials [32,33], but there has been no study on the preparation of a novel composite material by mixing gypsum with silica gel. Therefore, in previous studies, the author employed silica gel as a functional material to modify gypsum and prepare a gypsum–silica gel composite [34]. Through experimental measurements, the composite material exhibited markedly enhanced moisture absorption and desorption capacity as well as rate compared to pure gypsum. To further enhance the humidity-control capabilities of this composite material, we developed a novel gypsum-based humidity-control material. This was achieved by adding sepiolite powder activated by calcium chloride at a mass ratio of 20%, based on existing research [28,35]. The humidity-control performance of the material was then evaluated through experiments and simulated tests. The study aims to provide a new idea for continuously seeking low-cost and practical humidity-control building materials suitable for the construction field.

## 2. Materials and Methods

### 2.1. Materials

The study used gypsum material and calcium chloride from Sinopharm Chemical Reagent Co., Ltd., Shanghai, China. Silica gel was obtained from Henan Pubang Environmental Protection Materials Co., Ltd., Henan, China. Sepiolite powder was provided by Aladdin Biochemical Technology Co., Ltd., Shanghai, China. The preparation process of gypsum-based humidity-control material is described as follows.

#### 2.1.1. Preparation of Activated Sepiolite Powder

The sepiolite powder was placed in an air-drying oven (10HS, made in China) to dry at a temperature of 90 °C until the change in weight did not exceed 0.1% over three consecutive days. Subsequently, a 3 mol/L calcium chloride solution was formulated and blended with the dried sepiolite powder at a mass ratio of 8:2 with the aid of an electronic balance (TP-213, accuracy: ±0.01 g). The mixture was subsequently homogenized using a glass rod until it attained a dry and friable consistency to obtain sepiolite power activated by calcium chloride.

#### 2.1.2. Preparation of Gypsum–Silica Gel Humidity-Control Material

Firstly, the silica gel was crushed using a crusher (MT-10S, made in China) and sieved through a mesh to obtain 3 mm particle-size silica gel. Then, gypsum and silica gel were separately weighed in a mass ratio of 6:4 using an electronic balance (TP-213, accuracy: ±0.01 g). After thoroughly mixing the materials in a beaker, the desired amount of distilled water was added and stirred well. Finally, the well-mixed composite slurry was poured into a precast mold with dimensions of 50 × 50 × 5 mm and allowed to solidify. It was then cured for 24 h at a temperature of 50 ± 2 °C and a relative humidity of 40 ± 5%. This resulted in the formation of the gypsum–silica gel humidity-control material. The detailed preparation procedure can be found in reference [34].

#### 2.1.3. Preparation of Gypsum-Based Humidity-Control Material

The uncured gypsum–silica gel humidity-control material slurry was mixed with activated sepiolite powder in a mass ratio of 8:2. The mixture was poured into a beaker and the desired amount of distilled water was added, then stirred thoroughly to achieve uniformity. The well-mixed composite slurry was poured into a precast mold with dimensions of 50 × 50 × 5 mm and allowed to solidify. It was then cured for 24 h at a temperature of 50 ± 2 °C and a relative humidity of 40 ± 5%. This resulted in the formation of the gypsum-based humidity-control material.

**Figure 1:**
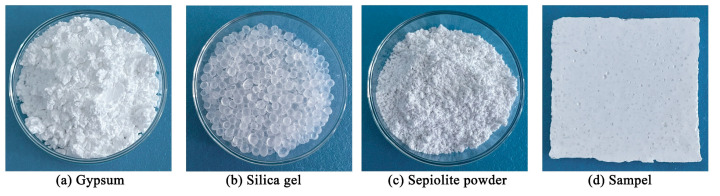
Raw materials and sample.

The raw materials used in the sample preparation process, as well as the samples employed during the experimental procedure, are illustrated in Figure 1.

The material composition parameters are shown in Table 1. The ratio of materials to water was determined based on the results of multiple preliminary experiments.

### 2.2. Testing Methods

#### 2.2.1. Experimental Testing

1. Humidity-control performance

To test the humidity-control performance of the gypsum-based humidity-control material, two experimental schemes were designed. First, the gypsum-based humidity-control material was used to dehumidify and humidify high-humidity (85%) and low-humidity (25%) environments consecutively. This continuous humidity-control test was repeated three times. Second, the gypsum-based humidity-control material was used to dehumidify the high-humidity environment (85%) and immediately humidify the low-humidity environment (25%). This intermittent humidity-control test was repeated three times.

**Figure 2:**
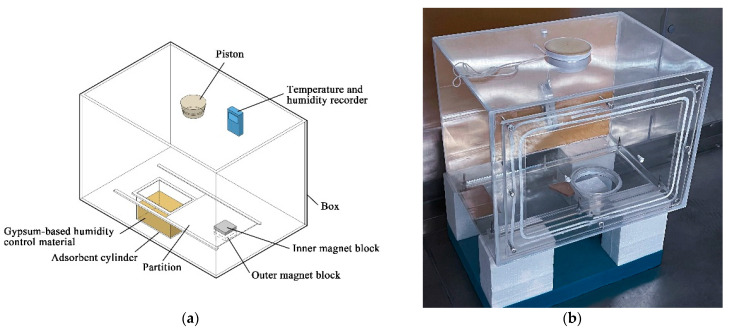
Experimental setup (**a**) design schematic diagram; (**b**) actual image.

The experimental setup design schematic diagram illustrated in Figure 2a consisted of a box and an adsorbent cylinder situated outside it. The outer magnet block slid along the outer guide rail, controlling the inner magnet block inside the box via magnetic attraction. This allowed for the opening or sealing of the partition between the box and the adsorbent cylinder as required. The experiment involved establishing high- or low-humidity conditions within the box by humidifying or dehumidifying through the piston port. After that, the dehumidification and humidification process was initiated by opening the sealing partition using the outer magnet block. In situ relative humidity measurements were performed using a temperature and humidity recorder (AZ8829, made in China) placed in the box every minute. The experimental temperature was maintained at 23 °C. The actual image of the experimental setup is shown in Figure 2b.

2. The impact of the environment on humidity-control performance

To test the effect of environmental temperature on the humidity-control performance of gypsum-based humidity-control material, the programmable temp&humi chamber (LY-2150B, made in China, accuracy: +2 °C; +3%RH) was utilized. A constant relative humidity of 80% and 20%, respectively, was maintained for the experiment. The materials were placed in an air-drying oven to dry at a temperature of 80 °C until the change in weight did not exceed 0.1% over three consecutive days. The dry materials were placed in the chamber with a constant relative humidity of 80% to absorb moisture. In contrast, the materials that had reached the maximum equilibrium moisture absorption at a relative humidity of 97.4% were placed in the chamber with a constant relative humidity of 20% to desorb moisture. The chamber was set to 13 °C, 23 °C, and 33 °C, respectively.

To test the effect of environmental relative humidity on the humidity-control performance of gypsum-based humidity-control material, the internal temperature of the programmable temp&humi chamber was kept at 23 °C. The dry materials were placed in the chamber to absorb moisture, and the relative humidity inside the chamber was controlled at a constant level of 50%, 60%, 70%, and 80%, respectively. The materials that had reached the maximum equilibrium moisture absorption at a relative humidity of 97.4% were placed in the chamber to desorb moisture. Inside the chamber, the relative humidity was controlled at constant levels of 20%, 30%, 40%, and 50%, respectively.

3. Isothermal moisture adsorption–desorption curve

In accordance with the Chinese standard GB/T 20312-2006 [36], the isothermal moisture adsorption–desorption curve was tested using the programmable temp&humi chamber method, and the internal temperature of the chamber was kept at 23 °C. The dry materials were placed in the chamber to absorb moisture. Inside the chamber, the relative humidity was controlled at constant levels of 22.5%, 32.9%, 43.2%, 53.5%, 64.9%, 75.4%, 84.6%, and 97.4%. The materials that had reached the maximum equilibrium moisture absorption at a relative humidity of 97.4% were placed in the chamber to desorb moisture. Inside the chamber, the relative humidity was controlled at a constant relative humidity of 84.6%, 75.4%, 64.9%, 53.5%, 43.2%, 32.9%, and 22.5%.

The equilibrium moisture content u during the process of moisture adsorption and desorption of the above material can be calculated using Formula (1),

u = (m − m_0_)/m_0_ (1)

where m_0_ is the dry mass of material (g), m is the mass of the material at moisture equilibrium (g).

The moisture content U during the process of moisture adsorption and desorption of the above material can be calculated using Formula (2),

U = (u_1_ − u_2_) ∗ 100 (2)

where u_1_ is the moisture content of the material after adsorption or before desorption (g/g), u_2_ is the moisture content of the material before adsorption or after desorption (g/g).

**Figure 3:**
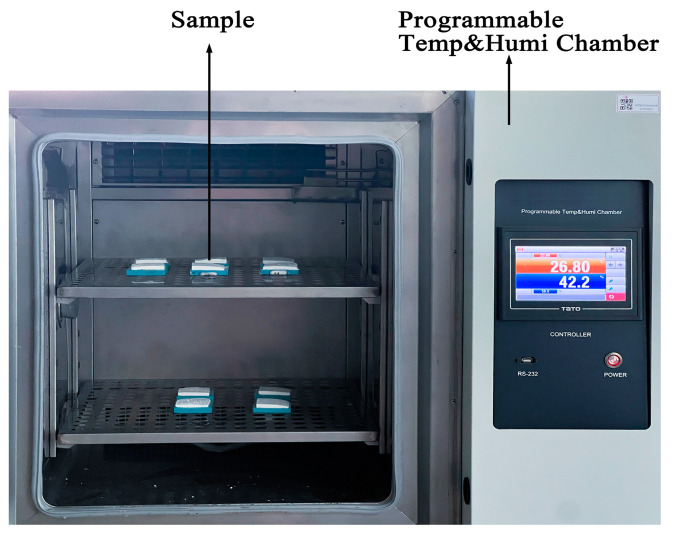
Experimental process of using the programmable temp&humi chamber.

The actual image showcasing the experimental process of using the programmable temp&humi chamber is illustrated in Figure 3.

#### 2.2.2. Simulation Testing

To test the humidity-control effect of gypsum-based humidity-control material applied in the building, EnergyPlus software (EnergyPlus 9.6.0) was used. The study focused on investigating the impact of material thickness and material laying area on the indoor air parameter changes in a room under summer climate conditions in Xiamen. The simulation period spanned from 1 August to 7 August.

To test the energy-saving effect of gypsum-based humidity-control material applied in buildings, EnergyPlus software was used. The study focused on investigating the impact of the laying and non-laying of material on building energy consumption under various climatic conditions. Four cities worldwide characterized by typical urban climates were selected for analysis. These cities include Beijing, China (continental monsoon climate), Paris, France (temperate oceanic climate), Atlanta, United States (subtropical and sub-humid climate), and Xiamen, China (subtropical oceanic monsoon climate).

**Figure 4:**
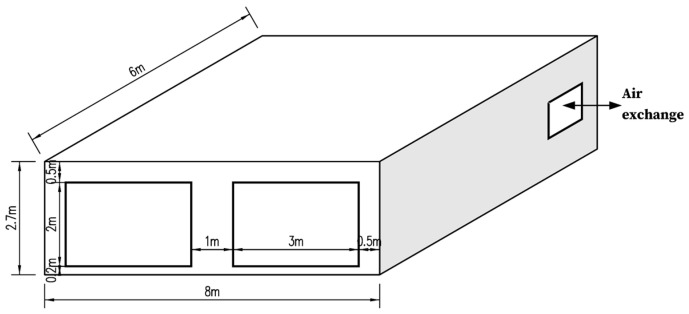
The BESTEST base case building.

For accurate and typical results, the BESTEST base case building (shown in Figure 4) from the IEA ECBCS Annex 21 was selected as the test building. The BESTEST base case is assumed to be a typical office house. The room is occupied during the daytime from 8:00 to 18:00 and unoccupied the rest of the day.

The detailed physical property parameters of each layer in the BESTEST base case building are presented in Table 2.

The simulation settings and boundary conditions are shown in Table 3.

Table 4 displays the materials utilized in the simulation. Case A studied the influence of gypsum-based humidity-control material thickness on its regulating humidity effectiveness. The thickness ranged from 0.01 m to 0.05 m, and the material laying area was 159.6 m^2^ (internal walls, floor, and ceiling). Case B studied the impact of gypsum-based humidity-control material laying area on its regulating humidity effectiveness. The material laying area was 63.6 m^2^ (internal walls) and 159.6 m^2^ (internal walls, floor, and ceiling), and the material thickness was 0.03 m. Finally, Case C acted as the reference group where no gypsum-based humidity-control material was applied in the room.

## 3. Results

### 3.1. Experimental Testing

#### 3.1.1. Humidity-Control Performance

**Figure 5:**
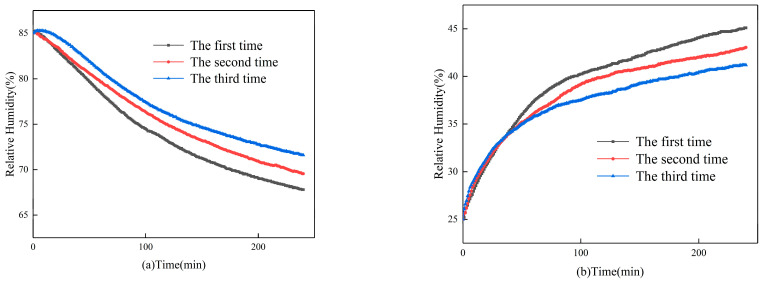
Results of (**a**) continuous dehumidification tests; (**b**) continuous humidification tests.

The results of the continuous dehumidification and humidification tests of the gypsum-based humidity-control material are shown in Figure 5. Figure 5a shows the results of continuous dehumidification tests. These results reveal that the dehumidification capacity of the material continuously decreases during three consecutive dehumidification tests. The first dehumidification reduces the relative humidity from 85% to 68%, the second dehumidification reduces it to 70%, and the third dehumidification only reduces it to 72%. Figure 5b presents the results of continuous humidification tests. These results demonstrate that the humidification capacity of the material exhibits a continuing downward trend during three consecutive humidification. The first humidification increases the relative humidity from 25% to 45%, the second humidification increases it to 43%, and the third humidification only increases it to 41%.

**Figure 6:**
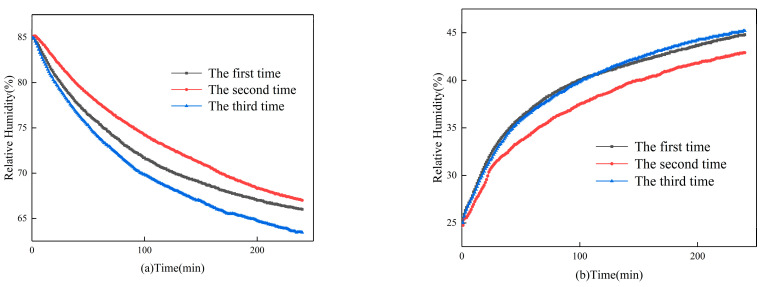
Results of (**a**) intermittent dehumidification tests; (**b**) intermittent humidification tests.

The results of the intermittent dehumidification and humidification tests of the gypsum-based humidity-control material are shown in Figure 6. Figure 6a shows the results of intermittent dehumidification tests. These results reveal that there is no attenuation in the dehumidification ability of the material during the three dehumidification tests. The relative humidity inside the box can be reduced from 85% to 70% in 130, 170, and 100 min for the first, second, and third dehumidification, respectively. Additionally, all three dehumidification tests achieve a stabilized relative humidity of 65% ± 2% within 200 min. Figure 6b shows the results of intermittent humidification tests. These results demonstrate that there is also no attenuation in the humidification ability of the material during the three humidification tests. The relative humidity can be increased from 25% to 40% in 100, 150, and 110 min for the first, second, and third humidification, respectively. Additionally, all three humidification tests can stabilize the relative humidity at 43% ± 2% within 200 min.

#### 3.1.2. The Impact of the Environment on Humidity-Control Performance

**Figure 7:**
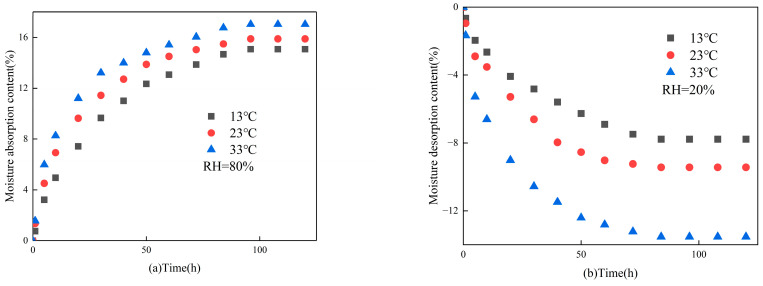
The impact of temperature on the (**a**) moisture absorption; (**b**) moisture desorption.

Figure 7 illustrates the absorption and desorption of the moisture content variations of gypsum-based humidity control under different ambient temperatures. As observed in Figure 7a, all dry materials under various ambient temperatures reached absorption moisture equilibrium after 100 h. Notably, there is a 2% increase in the equilibrium moisture absorption content when the ambient temperature changes from 13 °C to 33 °C. As observed in Figure 7b, all the materials that had reached the maximum equilibrium moisture absorption at a relative humidity of 97.4% under various ambient temperatures reached desorption moisture equilibrium after 100 h. The moisture desorption content also increases by 6%, when the ambient temperature increases from 13 °C to 33 °C. Observations demonstrate that alterations in ambient temperature minimally impact the moisture absorption content of the material. However, temperature fluctuations significantly affect the moisture desorption content of the material. The presence of silica gel in gypsum-based hygroscopic materials can account for this phenomenon. With increasing ambient temperature, the moisture absorption capacity of silica gel strengthens, consequently enhancing the moisture absorption capacity of the material.

**Figure 8:**
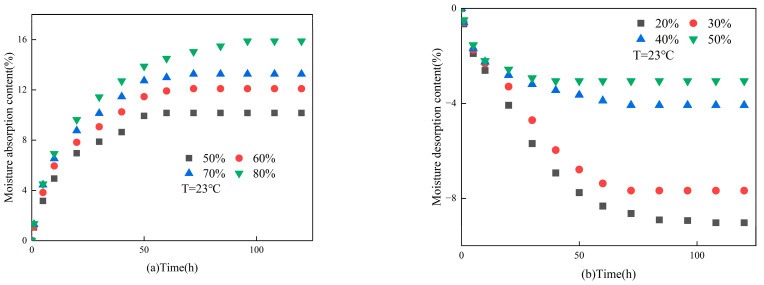
The impact of relative humidities on the (**a**) moisture absorption; (**b**) moisture desorption.

Figure 8 illustrates the absorption and desorption moisture content variations of gypsum-based humidity control under different ambient relative humidities. As depicted in Figure 8a, the material’s moisture absorption content was augmented by 6% upon an increase in relative humidity from 50% to 80%. This increase culminated at 15.88% at a relative humidity of 80%. As demonstrated in Figure 8b, the material’s moisture desorption content was augmented by 6% upon a decline in relative humidity from 50% to 20%. This increase culminated at 9.02% at a relative humidity of 20%. The experimental outcomes evince that the ambient relative humidity engenders an influence on both moisture absorption and desorption content of the material. Additionally, the greater the gap between the ambient relative humidity and the internal moisture content of the material, the more superior its performance in regulating moisture.

#### 3.1.3. Isothermal Moisture Adsorption–Desorption Curve

**Figure 9:**
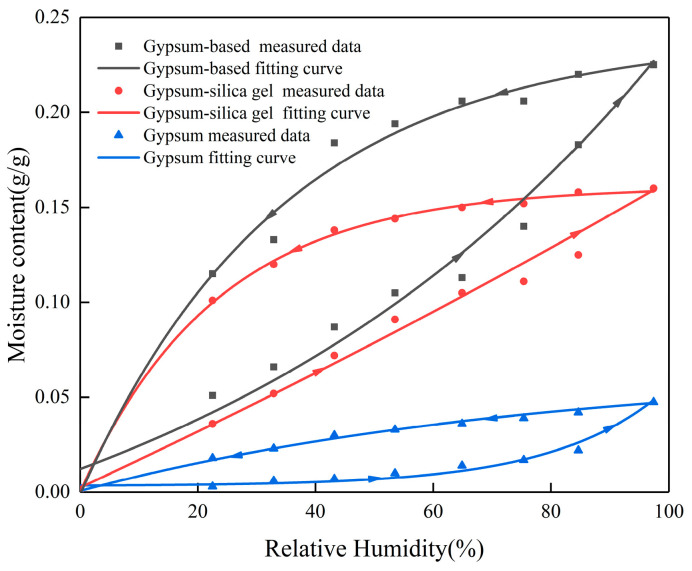
Isothermal moisture adsorption–desorption curve.

To evaluate and compare the humidity-control performance of pure gypsum, gypsum–silica gel humidity-control material, and gypsum-based humidity-control material, an isothermal moisture absorption and desorption test was conducted. Figure 9 indicated that the gypsum-based humidity-control material exhibited significantly superior moisture absorption and desorption performance compared to the other two materials. The humidity-control performance ranking was determined as follows: gypsum-based humidity-control material > gypsum–silica gel humidity-control material > pure gypsum. The maximum equilibrium moisture content of the gypsum-based humidity-control material was 0.225 g/g at 97.4% relative humidity. This value exceeded that of the gypsum–silica gel humidity-control material by 1.4 times and pure gypsum by 4.5 times. Incorporating sepiolite powder activated by calcium chloride resulted in further enhancement of the adsorption and desorption capacity of gypsum. This improvement led to better humidity-control performance compared to the gypsum–silica gel humidity-control material.

The ExpDec1 model in Origin software (Origin 2017) was used to fit the isothermal equilibrium moisture content curve, the formula is as follows,

y = y_0_ + A_1_e^−x/t1^ (3)

relevant parameters of the fitting curve are listed in Table 5.

### 3.2. Simulation Testing

#### 3.2.1. The Impact of Material Thickness on Indoor Air Parameters

**Figure 10:**
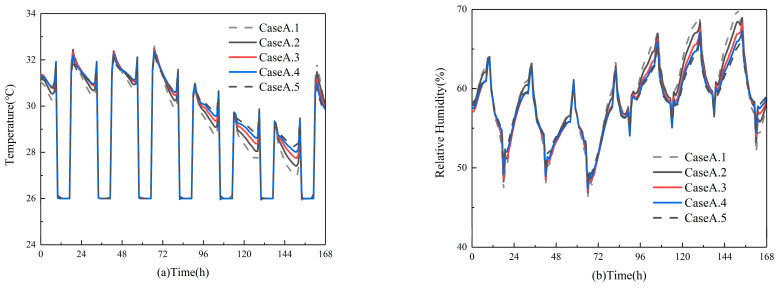
The impact of material thickness on (**a**) indoor temperature; (**b**) indoor relative humidity.

After conducting a simulated study on the impact of different thicknesses of materials laid on the interior of rooms under Xiamen’s summer climate conditions. These results are illustrated in Figure 10. The study found that the temperature trends of rooms with different thickness materials were similar. It was observed that as the thickness increased, the average indoor air temperature slightly increased. The average temperature of the room with a 0.05 m thickness material was 0.26 °C higher than that with a 0.01 m thickness material. Furthermore, the relative humidity in rooms with different thickness materials could be stabilized within the range of 40% to 70%. After laying the 0.05 m thick material, the change in indoor air relative humidity was the smallest and the trend was the most stable. However, there was not a significant difference compared to other thicknesses. The mean relative humidity of rooms with a 0.05 m thickness material was only 0.78% lower than rooms with a 0.01 m thickness material. In conclusion, the variation in material thickness has a negligible effect on indoor air parameters. This may be due to the large range of the laying area, and the change in material thickness almost does not affect the total amount of material used.

**Figure 11:**
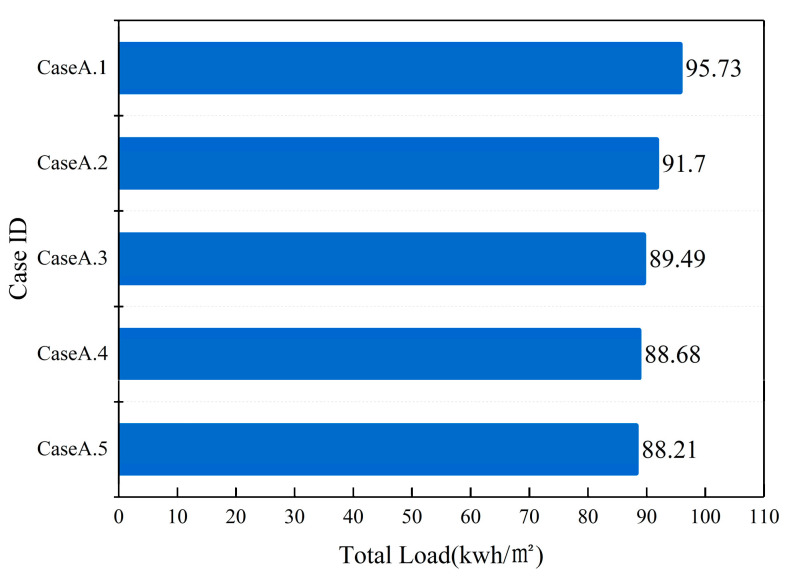
The impact of material thickness on energy-saving.

Based on the simulation results, the optimal thickness value of the material cannot be determined solely by changes in indoor air parameters. However, a specific thickness value needs to be determined in subsequent simulations. Figure 11 shows the energy consumption of the room with different thickness materials laid on the inside of the room. As apparent from Figure 11, as the laying thickness increases, energy consumption shows a significant downward trend. It is worth noting that when the laying thickness is 0.03 m, 0.04 m, and 0.05 m, the energy consumption is relatively close, at 89 ± 1 kwh/m^2^, which is significantly better than the energy consumption of 0.01 m and 0.02 m. Compared with laying thicknesses of 0.01 m and 0.02 m, energy efficiency has been improved by 7.25% and 3.17%, respectively. Considering economic factors, we choose 0.03 m as the laying thickness for subsequent simulations.

#### 3.2.2. The Impact of Material Area on Indoor Air Parameters

**Figure 12:**
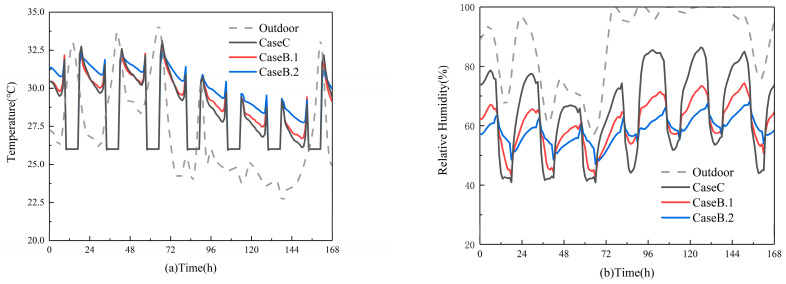
The impact of material area on (**a**) indoor temperature; (**b**) indoor relative humidity.

Then, a simulated study was conducted on the impact of different areas of materials laid on the interior of rooms on the indoor air parameters under Xiamen’s summer climate conditions. These results are illustrated in Figure 12. Figure 12a reveals that the average room temperature with laid material is higher than that without it, and the indoor temperature intensifies as the area of the material increases. This can be attributed to the exchange of outdoor heat into the indoor environment. It occurs due to high outdoor temperatures combined with strong sunlight, where the material retains some of the indoor heat load. As a result, there is an increase in the average indoor temperature. Figure 12b deduces that relative humidity outside remains high for most of the time. During the air conditioning operation period (8:00–18:00), both rooms exhibit comfortable levels of relative humidity. However, when the air conditioning is turned off, the relative humidity in the room without laid material may reach 80%, while the room with laid material can maintain indoor relative humidity at around 65%. This implies that the material has the potential to regulate indoor humidity fluctuations and stabilize indoor relative humidity within a narrow range as the area increases. Compared to the room without laid material and the room with a 63.6 m^2^ area, the room with a 159.6 m^2^ area exhibits a smaller amplitude of moisture content fluctuation.

#### 3.2.3. Analysis of Energy-Saving

Table 6 presents a comparative analysis of the annual energy consumption and energy-saving efficiency achieved by using gypsum-based humidity-control material in the interior of buildings located in different cities. These cities include Beijing, Paris, Atlanta, and Xiamen. The analysis reveals that the energy-saving efficiencies for these cities are 19.31%, 18.48%, 18.04%, and 10.81%, respectively. These findings demonstrate that the utilization of such material can effectively reduce building energy consumption, and their energy-saving effect is substantial. However, in Xiamen’s hot and humid climate, it should be noted that high outdoor temperatures elevate the thermal load accumulated by the material. Additionally, the outdoor relative humidity impedes the desorption process of the material, leading to suboptimal discharge of internal moisture. Consequently, in areas with large temperature and relative humidity fluctuations throughout the year, such as Beijing, Paris, and Atlanta, the materials manifest excellent energy-saving capabilities. This suggests that if the heat and moisture retained by the material are expelled promptly, and the heat dissipation and desorption processes are facilitated, the energy-saving performance of the material will improve.

## 4. Discussion

Through the analysis of experimental results, it was found that intermittent dehumidification and humidification have shown more beneficial long-term performance for the material compared to continuous dehumidification and humidification. Moreover, although high temperature can promote the desorption process of gypsum-based humidity-control material, its desorption capacity is still limited under continuously high-humidity conditions. In contrast to temperature, relative humidity has a more significant impact on the material’s humidity-control performance.

The simulation results also confirmed the findings. In climates characterized by sustained high temperature and humidity, such as Xiamen, outdoor high temperature increases the accumulated heat load on the material. Additionally, outdoor high humidity hinders the desorption process, resulting in suboptimal discharge of internal moisture. Conversely, in regions with greater fluctuations in temperature and relative humidity throughout the year, such as Beijing, Paris, and Atlanta, the material exhibits excellent energy-saving performance.

**Figure 13:**
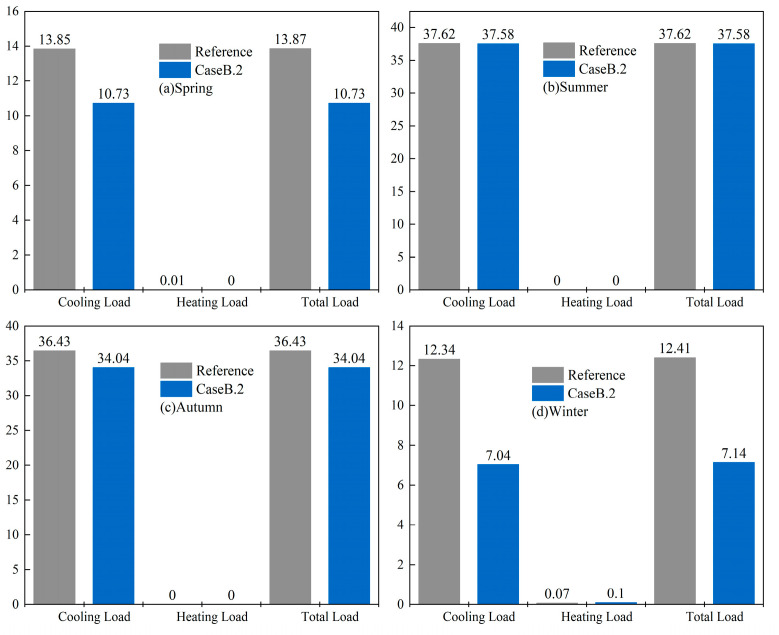
Energy consumption of Xiamen office buildings in (**a**) spring, (**b**) summer, (**c**) autumn, and (**d**) winter.

To better elucidate this finding, Xiamen was taken as an example to categorize the impact of gypsum-based humidity-control material on annual building energy consumption according to the seasons: spring, summer, autumn, and winter. The results are presented in Figure 13. During spring, it can be observed that there is a large temperature and relative humidity difference between daytime and nighttime. The temperature is higher during the day, and the relative humidity is lower. Under this circumstance, the material can absorb the heat from the surrounding environment by releasing the moisture stored in the internal capillary pores, thereby effectively reducing the indoor temperature. Conversely, at night, the temperature drops, and the relative humidity increases, allowing the material to absorb moisture from the air that condenses inside the material and releases heat, leading to an increase in indoor temperature. Consequently, this material exhibits excellent energy-saving performance during spring. During summer, due to the continuous high temperature and humidity in Xiamen, the internal waste heat and moisture are not fully discharged and released. As a result, this limits the energy-saving capacity of the material. Similarly, during autumn, although outdoor temperature and humidity decrease relative to summer, they remain relatively high overall. This indicates that the material cannot efficiently discharge excess internal heat and moisture, thereby resulting in weak energy-saving performance. During winter, it exhibits similar characteristics to spring, with significant diurnal temperature variation and relative humidity. However, the average temperature and average relative humidity are the lowest throughout the year, with an average temperature of 17.43 °C and an average relative humidity of 58.3%. During the daytime, the temperature is relatively high, and the humidity is lower. This creates an environment where the material can release moisture from its pores, effectively reducing the indoor temperature and decreasing the cooling load on air conditioning. However, as the nighttime temperature drops, the remaining moisture may further lower the indoor temperature, increasing the heating load on air conditioning. Overall, the material still demonstrates good energy-saving effects in winter.

## 5. Conclusions

To improve the humidity-control performance of traditional building materials and augment their efficacy in regulating indoor relative humidity, this study incorporated sepiolite powder activated by calcium chloride into gypsum–silica gel humidity-control material to prepare a new type of gypsum-based humidity-control material. Through experimental and simulation studies, the following main conclusions were drawn:

(1) Gypsum-based humidity-control material exhibits the ability to absorb moisture in high-humidity environments and desorb moisture in low-humidity environments. However, the material’s humidity-control capacity decreases over time during continuous dehumidification or humidification. Therefore, intermittent dehumidification and humidification are more conducive to maintaining relative humidity stability for extended periods of time.

(2) The moisture absorption content of the gypsum-based humidity-control material is minimally influenced by changes in environmental temperatures, while its moisture desorption content is noticeably affected. On the other hand, variations in relative humidity impact both the moisture adsorption and desorption content of the material. The larger the discrepancy between environmental relative humidity and the internal moisture content of the material, the greater the effectiveness of the material’s humidity-control performance.

(3) Addition of calcium-chloride-activated sepiolite powder significantly enhances the adsorption and desorption abilities of the gypsum–silica gel humidity-control material. At a relative humidity of 97.4%, the equilibrium moisture content of the gypsum-based humidity-control material can reach a maximum of 0.225 g/g. It is 1.4 times higher than that of gypsum–silica gel material, and 4.5 times than that of pure gypsum materials.

(4) Gypsum-based humidity-control material can mitigate the influence of exterior and interior humidity loads on indoor relative humidity. The material can reduce fluctuations in indoor relative humidity and maintain it within a narrow range, providing a more stable indoor environment.

(5) Gypsum-based humidity-control material has the potential to reduce building energy consumption. Simulation results show that compared with regions with high temperatures and high humidity throughout the year, this material is more suitable for areas with large diurnal temperature differences and differences in relative humidity.

The uniqueness of this study lies in the successful preparation of a novel gypsum-based humidity-control material. It provides a new approach for low-cost and practical humidity-control building materials in the field of architecture. However, there are still several limitations to be considered. This study explores the practical application of gypsum-based humidity-control materials through simulation research methods. Further work requires long-term monitoring and analysis in actual architectural environments to evaluate their actual moisture regulation performance. Additionally, during the preparation process of gypsum-based composite materials, the silica gel and activated sepiolite powder as additives significantly enhance the moisture absorption and desorption properties of the materials. However, considering that silica gel itself belongs to a “strong absorption and weak desorption” material, it leads to relatively poor desorption performance of gypsum-based materials. The next focus of research will concentrate on exploring modification methods to improve the moisture desorption performance of the materials. Simultaneously, it aims to achieve cost reduction objectives and ensure meet energy-saving requirements in continuous high-temperature and high-humidity environments.

## Figures and Tables

**Table 1:** Materials composition parameters.

Material	Material Mass Ratio	Material to Water Mass Ratio
activated sepiolite power	80% sepiolite power + 20% calcium chloride	-
gypsum–silica gel material	60% gypsum + 40% silica gel	1:0.7
gypsum-based material	80% gypsum–silica gel material + 20% activated sepiolite power	
gypsum material	100% gypsum	

**Table 2:** Physical parameters of different layers in building envelopes.

Construction	Material	d (m)	$\rho \;\;(\mathrm{kg}/m^{3})$	$c_{p}\;\;(J/\mathrm{kg}\cdot K)$	K (W/m·K)	U (W/m^2^·K)
Wall (from outer layer to inner layer)	Wooden board	0.01	530	900	0.14	0.474
	Rock wool board	0.066	60	850	0.04	-
	Concrete	0.1	1400	1000	0.51	-
	gypsum-based material	-	1000	2250	0.45	-
Roof (from outer layer to inner layer)	Cement panel	0.012	1130	840	0.255	0.307
	Rock wool board	0.066	60	850	0.04	-
	gypsum-based material	-	1000	2250	0.45	-
Floor (from outer layer to inner layer)	Thermal insulating	1	60	850	0.04	0.04
	layer	-	-	-	-	-
	gypsum-based material	-	1000	2250	0.45	-
Window	Double glazing unit	-	-	-	-	1.99

**Table 3:** Simulation settings and boundary conditions.

Conditions	Office Case
Occupied period	8:00–18:00
Unoccupied period	The rest of the day
Air conditioning running time	Occupied period
Air conditioning running temperature (°C)	18–26
Air conditioning running relative humidity (%)	30–70
Air change rate (ACH)	0.5
Air infiltration	No

**Table 4:** Simulating material.

Case ID	Thickness (m)	Area (m^2^)
Case A.1	0.01	159.6
Case A.2	0.02	
Case A.3	0.03	
Case A.4	0.04	
Case A.5	0.05	
Case B.1	0.03	63.6
Case B.2		159.6
Case C	-	0

**Table 5:** Parameters of sample isothermal equilibrium moisture content fitting curve.

	Parameters	Gypsum-Based Material	Gypsum–Silica Gel Material	Gypsum
Absorption process	y_0_	−0.08307	−0.654	0.00327
	A_1_	0.09532	0.65651	2.5 × 10^−4^
	t_1_	−82.5884	−455.15	−18.7285
	R^2^	0.97	0.98	0.97
Desorption process	y_0_	0.24001	0.16084	0.06589
	A_1_	−0.24108	−0.16068	−0.06519
	t_1_	34.41	23.24	78.49
	R^2^	0.99	0.99	0.98

**Table 6:** Annual energy consumption and energy saving of different cities in office buildings.

City	Load and Efficiency	Case ID	
		Reference	CaseB.2
Beijing	Total load (kwh/m^2^)	77.88	62.84
	Energy saving (%)	-	19.31
Paris	Total load (kwh/m^2^)	60.77	49.54
	Energy saving (%)	-	18.48
Atlanta	Total load (kwh/m^2^)	112.13	91.9
	Energy saving (%)	-	18.04
Xiamen	Total load (kwh/m^2^)	100.34	89.49
	Energy saving (%)	-	10.81

## Data Availability

Data sharing not applicable.
